# Pseudo-chaotic oscillations in CRISPR-virus coevolution predicted by bifurcation analysis

**DOI:** 10.1186/1745-6150-9-13

**Published:** 2014-07-02

**Authors:** Faina S Berezovskaya, Yuri I Wolf, Eugene V Koonin, Georgy P Karev

**Affiliations:** 1 Howard University, 6-th Str, Washington, DC 20059, USA; 2 National Center for Biotechnology Information, National Library of Medicine, National Institutes of Health, Bethesda, MD 20894, USA

## Abstract

**Background:**

The CRISPR-Cas systems of adaptive antivirus immunity are present in most archaea and many bacteria, and provide resistance to specific viruses or plasmids by inserting fragments of foreign DNA into the host genome and then utilizing transcripts of these spacers to inactivate the cognate foreign genome. The recent development of powerful genome engineering tools on the basis of CRISPR-Cas has sharply increased the interest in the diversity and evolution of these systems. Comparative genomic data indicate that during evolution of prokaryotes CRISPR-Cas loci are lost and acquired via horizontal gene transfer at high rates. Mathematical modeling and initial experimental studies of CRISPR-carrying microbes and viruses reveal complex coevolutionary dynamics.

**Results:**

We performed a bifurcation analysis of models of coevolution of viruses and microbial host that possess CRISPR-Cas hereditary adaptive immunity systems. The analyzed Malthusian and logistic models display complex, and in particular, quasi-chaotic oscillation regimes that have not been previously observed experimentally or in agent-based models of the CRISPR-mediated immunity. The key factors for the appearance of the quasi-chaotic oscillations are the non-linear dependence of the host immunity on the virus load and the partitioning of the hosts into the immune and susceptible populations, so that the system consists of three components.

**Conclusions:**

Bifurcation analysis of CRISPR-host coevolution model predicts complex regimes including quasi-chaotic oscillations. The quasi-chaotic regimes of virus-host coevolution are likely to be biologically relevant given the evolutionary instability of the CRISPR-Cas loci revealed by comparative genomics. The results of this analysis might have implications beyond the CRISPR-Cas systems, i.e. could describe the behavior of any adaptive immunity system with a heritable component, be it genetic or epigenetic. These predictions are experimentally testable.

**Reviewers’ reports:**

This manuscript was reviewed by Sandor Pongor, Sergei Maslov and Marek Kimmel. For the complete reports, go to the Reviewers’ Reports section.

## Background

The arms races between microbes and viruses preying on them often display rich, complex population dynamics [1]. In principle, the dynamics of virus-microbe interactions is analogous to the classical predator–prey models [2-5] but both microbes and viruses evolve much faster than animals such that virus-host interactions change on a scale that may be amenable to direct laboratory study. One of the adaptation mechanisms employed by hosts to curb viruses is the CRISPR-Cas (*C*lustered *R*egularly *I*nterspaced *S*hort *P*alindromic *R*epeats-*C*RISPR *as*sociated proteins), a recently discovered adaptive immunity system that is present in the great majority of Archaea and many bacteria [6-12]. Microbes create heritable memory of viruses that attack them by inserting virus-derived spacers into CRISPR repeat cassettes, thus following the Lamarckian modality of evolution that dramatically accelerates adaptation [13]. The rapid adaptation through the activity of CRISPR-Cas is possible because this system engenders heritable genetic changes that are directly beneficial for the archaeon or bacterium in the face of a specific environmental challenge (a virus), in contrast to the random, undirected mutations in the Darwinian evolutionary framework [14]. The CRISPR-Cas systems are increasingly used as powerful, versatile tools for genomic engineering tools which sharply increases the interest in their diversity and evolution [15-20].

General considerations suggest that the population dynamics of virus-host coevolution should be dominated by periodic selective sweeps alternating between the host (when it "discovers" a resistance mutation or acquires immunity against the dominant virus lineage) and the virus (when an immunity escape mutation occurs), similar to the case of rapidly evolving human viruses [21]. Indeed, such behavior has been observed in simple Lotka-Volterra type models of phage-bacteria coevolution [22,23] as well as in direct evolutionary experiments [23]. However, direct and indirect population studies reveal much more complex behaviors of the actual populations that usually does not involve strain dominance and instead displays long-term persistence of multiple lineages of both the microbial hosts and the viruses [24-27].

Several detailed agent-based models of coevolution between viruses and CRISPR-Cas-carrying hosts have been developed and analyzed [26,28-35]. The agent-based models incorporate the salient features of the CRISPR-Cas system such as the existence of the CRISPR cassette with virus-derived spacers, immunity conferred to a host by spacers that match the attacking virus and acquisition of new spacers as a result of failed virus infections. These models allow one to reproduce many aspects of the observed behavior of coevolving virus-host systems and predict conditions required for the evolutionary maintenance of the CRISPR-Cas immunity, such as a threshold of viral diversity [28].

Agent-based models provide for the exploration of interactions of arbitrary complexity and naturally incorporate the desired level of granularity (e.g. individual-based or lineage-based models) and the stochasticity of the processes involved. However, such models typically possess a high-dimensional parameter space that cannot be explored in full, so that not all potential regimes, some of which could be biologically relevant, are captured. In contrast, mathematical models based on systems of differential equations are limited in complexity and are inherently less realistic (at the very least because they approximate reality with infinitely small deterministic changes) but when analytically tractable, permit a full and rigorous analysis of all possible behaviors.

Here we describe Lotka-Volterra type models of interaction between a host with a heritable adaptive immunity system, such as CRISPR-Cas, and a virus that escapes the immunity via implicit accumulation of mutations which is implemented as gradual immunity decay. We construct "minimal" analytical models which capture qualitatively the basic regimes of the CRISPR-Cas system behaviors previously found experimentally and through the agent-based modeling. We explore the full spectrum of possible behaviors of this virus-host system, compare the results with those of a more detailed agent-based model [32], and describe a previously unnoticed regime of quasi-chaotic oscillations.

## Results

### Three-component CRISPR population dynamics: Malthusian and logistic versions

In order to construct "minimal" analytical models that would qualitatively capture the basic regimes of the CRISPR-Cas system behaviors we analyzed, as a preliminary step, a two-component Volterra-type model which describes the dynamics of virus-host system and consider immunity static within the timescale of the model. Our analysis shows that two-component models display simple dynamical regimes (see Methods for details). However, the CRISPR-Cas system of adaptive immunity, which this work seeks to model, cannot be expected to follow these simple regimes given its dynamic evolution that involves rapid acquisition of immunity to a particular virus or plasmid along with loss and gain of entire CRISPR-Cas loci [36]. Thus, more realistic models should take into account that adaptive immunity systems are characterized by the existence of immune memory that enhances the response in individuals encountering a familiar challenge [37,38]. Originally non-immune ("native") individuals can acquire the adaptive response capacity that persists at timescales relevant for our model. Thus, we introduce two categories of hosts, immune and non-immune, that interact differently with the virus and exchange individuals via immunity acquisition and decay.

Let *x*(*t*) be the density of immune hosts with the immunity *p* with respect to viruses, 0 ≤ *p* ≤ 1; *y*(*t*) be the density of sensitive hosts with immunity *s,* 0 ≤ *s* ≤ *p* ≤ 1; *z*(*t*) be the density of viruses. We consider the following 3-component model:

(1) $$\begin{array}{l} \frac{\text{dx}}{\text{dt}}=x(1-l-a\left(x+y\right))-\text{bxz}(1-p)+\text{esyz}\equiv P(x,y,z),\\ \frac{\text{dy}}{\text{dt}}=y+\mathrm{lx}-\text{ay}\left(x+y\right)-\text{byz}(1-s)-\text{esyz}\equiv Q(x,y,z),\\ \frac{\text{dz}}{\text{dt}}=z(-d+\text{bM}(x\left(1-p\right)+y(1-s))-b(\text{xp}+\text{ys}))\equiv R(x,y,z). \end{array}$$

Here *l* is the immunity decay rate (*x* → *y* flow), *e* is the immunity acquisition rate; *d* is the death rate of viruses, *M* is the virus reproduction rate;  *b* is the encounter rate coefficient; the growth rates of immune and sensitive hosts are equal to 1.

Model (1) for *a* = 0 describes a situation when immune and sensitive hosts in the absence of viruses grow according to the Malthusian model. If *a* > 0, the model (1) describes a more realistic situation when both classes of hosts grow according to the logistics model.

Coordinates of equilibria of (1) solve the system:

(2) $$\begin{array}{l} P\left(x,y,z\right)\equiv x(1-l-a(x+y))-\text{bxz}(1-p)+\text{esyz}=0,\\ Q\left(x,y,z\right)\equiv y+\text{lx}-\text{ay}(x+y)-\text{byz}(1-s)-\text{esyz}=0,\\ R\left(x,y,z\right)\equiv z(-d+\text{bM}(x(1-p)+y(1-s))-b(\text{xp}+\text{ys}))=0. \end{array}$$

Model (1) has equilibrium *O*(0, 0, 0) in all cases. The logistic model with *a* > 0  has one more equilibrium *A*(0,1/*a*,0), which corresponds to existence of only non-immune hosts.

In Methods the following assertions are proven.

Statement 1.

(1) *Equilibrium O*(*x* = *y* = *z* = 0) *is unstable for all parameter values;*

(2) *If a* > 0 *then equilibrium A(0,1/a,0) is stable for*$M<\frac{\text{da}+\text{bs}}{b\left(1-s\right)}$*and unstable for*$M>\frac{\text{da}+\text{bs}}{b\left(1-s\right)}.$

If the immunity *p* in model (1) is a constant, then, additionally, the model may have either non-trivial stable equilibrium, or may demonstrate periodic oscillations. Detail description of possible behaviors of the model with constant *p* is given in Methods.

More realistically, the immunity *p* is not a constant but depends on the density of the virus, *p* = *p*(*z*) (0 ≤ *p* ≤ 1). Below we consider the latter case, in agreement with empirical observations and computer simulations. Specifically, it has been shown that CRISPR-Cas systems are (nearly) ubiquitous in archaeal and bacterial hyperthermophiles are present in less than half of the available mesophile genomes [28,32,39,40]. Analysis of agent-based models of virus-host coevolution suggest that this distinction stems from the fact that hyperthermophiles face lower virus loads and diversity than mesophiles providing for higher efficacy of CRISPR-Cas [28].

Our aim is to find all stable modes of the model at different values of the model parameters and to describe the transitions from one mode to another when parameters vary; by other words, we want to construct the bifurcation diagram of the model. It is natural to suppose that *p* = *p*(*z*) monotonically decreases and tends to the immunity *s* of sensitive hosts at large *z*. From now on we consider:

(3) $$p\left(z\right)=\left(1-s\right)e^{-\text{kz}}+s$$

where *k, s* are constants, 0 < *s <* 1, *k >* 0*.* Under equation (3), immunity is a monotonically declining function of the virus amount that tends to a constant, maximum *p* (maximally efficient adaptive immunity) when *z* tends to zero, and tends to *s* (no adaptive immunity, innate immunity only) when *z* tends to infinity.

Let us consider model (1) with the immunity *p* = *p*(*z*) defined by (3). We do not attempt a complete analysis of this model but rather seek to identify stable modes and most interesting dynamical behaviors.

We start with the Malthusian version when *a =* 0. It has non-trivial equilibrium *B*_*e*_(*x*_*e*_, *y*_*e*_, *z*_*e*_) such that *x* = *x*_*e*_, *y* = *y*_*e*_ are expressed via *z* = *z*_*e*_:

(4) $$x_{e}=\frac{-d\left(1-b\left(1-s\right)z\right)}{b\left(p-s\right)\left(1+M-\text{bz}\right)},\;y_{e}=\frac{d\left(1-b\left(1-p\right)z\right)}{b\left(p-s\right)\left(1+M-\text{bz}\right)},$$

where *z* = *z*_*e*_ solves the equation

(5) $$\begin{array}{l} \left(1-l\right)+b\left(-2+l+p+s-\text{es}\left(1-l\right)\right)z+b^{2}\left(1-p\right)\\ \;\times \left(1-s+\text{es}\right)z^{2}=0. \end{array}$$

In particular, we show that for a wide domain of the parameter values, the model demonstrates non-periodic oscillation of all three variables. The following assertions are valid (see Methods).

**Statement 2.** *For a wide range of (fixed) parameters l*, *e*, *s*, *b*, 0 < *k* ≤ 1, *system (1), (3) with a = 0 has only one positive and unstable equilibrium B*_*e*_(*x*_*e*_, *y*_*e*_, *z*_*e*_) *under condition*p(ze)<MM+1;*the coordinates* (*x*_*e*_, *y*_*e*_, *z*_*e*_) *of this equilibrium satisfy (4), (5).*

**Figure 1:**
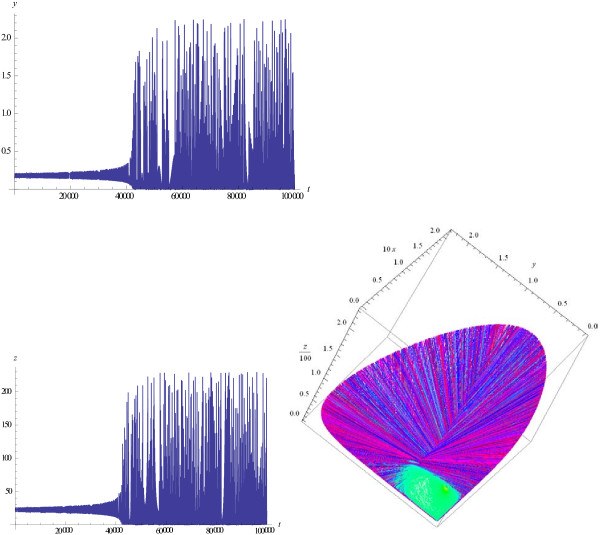
**Solutions {*x *(*t*),** ***y*(*t*),** ***z*(*t*)} and phase portrait of the Malthusian model (1), *a =*** **0 with parameter values *l* = 0.9, *e*** **= 0.1.** Other parameters are *M* = 100, *d* = 1, *b* = 0.01 , *k* = 0.5, *s* = 0.1. Initial values *x*(0) = 0.07, *y*(0) = 0.15, *z*(0) = 22.25 are chosen to be close to the equilibrium.

Trajectories of the model show quasi-chaotic behavior for a broad range of the model parameters. Consider in detail the behavior of the model solutions depending on the values of the parameters *l*, *e*, and *M*. For *l* > *e*, typical trajectories starting close to the equilibrium point are shown at Figure 1. Initially, all variables show almost periodic oscillations with increasing amplitudes. Then, as the amplitudes become large enough, the behavior of the trajectories changes sharply and the oscillations become (quasi)-chaotic; if the initial point is far from the equilibrium, then the (quasi)-chaotic oscillations are observed from the very beginning. When *l* < *e* the behavior of the trajectories is similar. The difference is that the fraction of immune hosts, *x*, in case *l* < *e* is greater than it is in case *l* > *e*. Again, if the initial point is taken far from the equilibrium, then the (quasi)-chaotic oscillations are observed from the very beginning, similar to Figure 1. These types of behavior are observed in a wide area of values of the parameter *M*, 1 < *M* < 1000. Notice, that when *M* increases, the maximum values of *x,y* decrease whereas *z* does not depend on *M*. This effect does not seem to have a plausible biological interpretation (viruses cannot exist if the hosts go extinct), indicative of apparent limitations of the model.

Let us consider a more realistic version of 3-component system (1) with logistic growth of hosts and the immunity *p*(*z*) given by (3). Again, we do not attempt a complete analysis of this model but rather seek to identify stable modes and most interesting dynamical behaviors and to compare them to those of the Malthusian version of the model. In particular, we show that the model displays non-trivial stable equilibria, stable oscillations, and quasi-chaotic oscillations of all three variables. More precisely, there exists a critical value of the virus birth rate *M*^*cr*^ at fixed values of all other parameters such that the system tends to a stable equilibrium when *M* < *M*^*cr*^, but if *M* > *M*^*cr*^, then small periodic oscillations appear due to the Hopf bifurcation and then, if *M* > > *M*^*cr*^, the system transits to a regime of (quasi)-chaotic oscillations.

Coordinates of any non-trivial equilibrium *B*(*x*_*e*_, *y*_*e*_, *z*_*e*_) should satisfy system (2); taking *a* = 1 for simplicity, we present system (2) in the form:

(6) $$\begin{array}{l} \left(x+y\right)-(x+y){}^{2}-b(x+y)z+b(\text{px}+\text{sy})z=0,\\ -d+\text{bM}\left(x+y\right)-b(\text{px}+\text{sy})(M+1)=0,\\ y+\text{lx}-y\left(x+y\right)-b(1-s+\text{es})\text{yz}=0,\\ p=p\left(z\right)=(1-s)\text{exp}(-\text{kz})+s \end{array}$$

It follows from the first two equations of (6) that

$${\left(x+y\right)}^{2}-\left(x+y\right)\frac{\left(1+M-\text{bz}\right)}{M+1}+\frac{\text{dz}}{M+1}=0.$$

The solution to the last equation is $\;x+y=\frac{1+M-\text{bz}+\sqrt{{\left(1+\text{bz}-M\right)}^{2}-4d\left(1+M\right)z}}{2\left(1+M\right)}\leq \frac{1+M-\text{bz}}{1+M}.$ So,

(7) $$\begin{array}{l} 0\leq x_{e}+y_{e}<1\;\text{and}\;z_{e}<\frac{1+M}{b}. \end{array}$$

It means that all possible non-trivial equilibria of the model are placed in a bounded area of the variable values; the amount of hosts is comparatively small (<1) and the amount of viruses is restricted by the virus reproduction rate *M*.

Solving system (6) we find coordinates of non-trivial equilibrium *B*(*x*_*e*_, *y*_*e*_, *z*_*e*_) such that *x* = *x*_*e*_, *y* =  *y*_*e*_ are expressed via *z* = *z*_*e*_

(8) $$\begin{array}{l} x=\frac{-d+bf^{\pm }\left(z\right)\left(M\left(1-s\right)-s\right)}{b\left(1+M\right)\left(p-s\right)},\\ y=\frac{d-bf^{\pm }\left(z\right)\left(M\left(1-p\right)-p\right)}{b\left(1+M\right)\left(p-s\right)},\text{where}\\ f^{\pm }\left(z\right)=\frac{1+M-\text{bz}\pm \sqrt{{\left(1+M-\text{bz}\right)}^{2}-4\text{ad}\left(1+M\right)z}}{2a\left(1+M\right)} \end{array}$$

and *z* -coordinate solves the equation:

(9) $$\begin{array}{l} \left(-1+l+af^{\pm }\right)(d-bf^{\pm }(M(1-s)-s))\\ \;+(\text{des}-b^{2}f^{\pm }(1-p)(M(1-s)-s)+b(d\left(1-p\right)\\ \;-ef^{\pm }(M(1-p)-p)s))z=0. \end{array}$$

Analysis of the system (1) with *a >* 0, (3) showed that there exists such threshold *M*^*cr*^ (depending on the model parameters) that the equilibrium *B* is stable if *M* < *M*^*cr*^; when *M* increases and intersects the threshold *M* = *M*^*cr*^, the equilibrium *B* loses stability and a stable limit cycle appears in the system. We summarize the results of this analysis in

**Statement 3.** *For a wide range of (fixed) parameters l*, *e*, *s*, *b*, 0 < *k* ≤ 1, *model (1), (3) with a = 1 has a stable equilibrium for* 0 < *M* < *M*^*cr*^*and stable oscillations for M* > *M*^*cr*^, *which appear due to supercritical Hopf bifurcation at M* = *M*^*cr*^(*l*, *e*, *s*, *b*).

See Methods for sketch of the proof.

**Figure 2:**
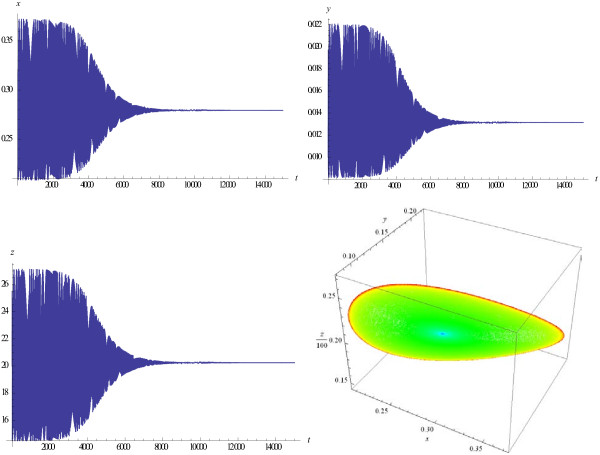
**Solutions {*x*(*t*),** ***y*(*t*),** ***z*(*t*)} and phase portrait of the logistic model (1), (3).** Trajectories tend to the stable equilibrium *B*(0.279, 0.013, 20.24); *M* = 98.225 < *M*^*cr*^; *k* = 0.1, *s* = 0.2, *b* = 0.05, *l* = 0.1, *e* = 0.5.

**Figure 3:**
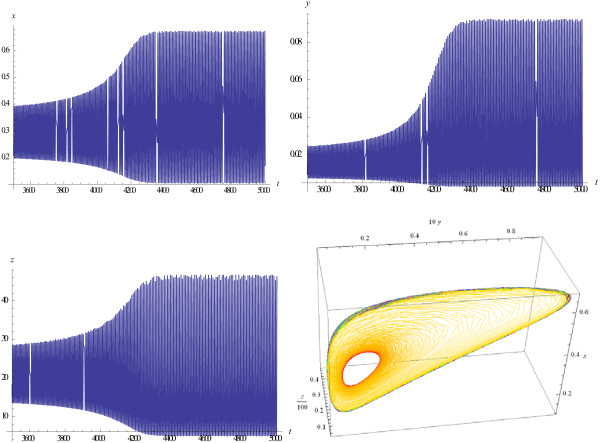
**A limit cycle appears in the system (1), (3);** ***M*** **= 98.226 >** ***M***^***cr***^**.**

**Figure 4:**
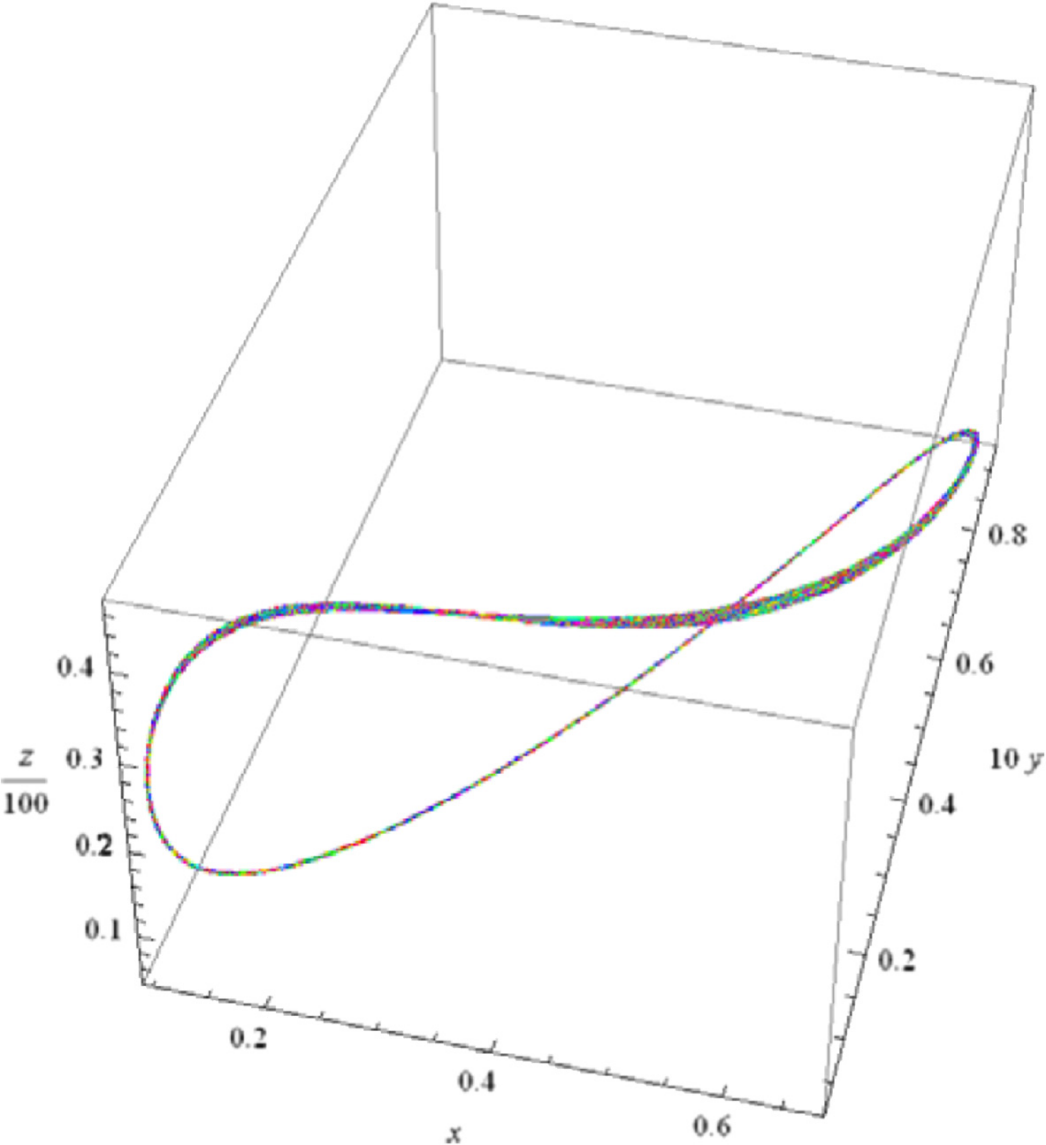
**The limit cycle, phase curve; 5000 <** ***t*** **< 30000, M = 98.226.**

**Figure 5:**
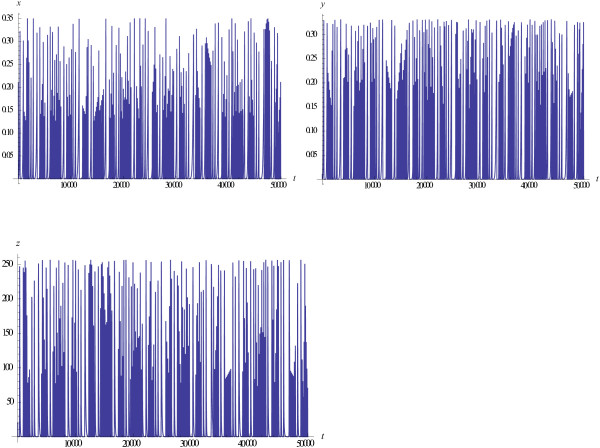
Trajectories show quasi-chaotic oscillations, phase curves fill a surface; M = 500.

Examples of the evolution of trajectories and phase portraits as the value of *M* increases are given in Figures 2, 3, 4, 5. Figure 2 shows trajectories of the system that tend to a stable equilibrium when the parameter *M* is below the bifurcation threshold. Figure 3 demonstrates that the system arrives at a stable limit cycle when *M* intersects the bifurcation threshold, and Figure 4 shows that the established regime at the steady state is very close to periodic oscillations.

Oscillations that are observed in Figure 4, with *M* above the bifurcation threshold *M*^*cr*^, are very close to but not perfectly periodic. This peculiarity of the trajectories can be explained in the following way. The Hopf theorem for a 3-dimension system states (see, e.g., [41], ch.5) that there exists such one-to-one transformation of the initial variables *x* → x*, *y* → y*, *z* → z* that two of new variables (say, x*  and  y*) show stable periodic oscillations but the third one does not and is governed by a separate equation. For this reason, the trajectories of initial variables, which are functions of x*, y*, z*, may be non-periodic and may show not exactly periodic and even quasi- chaotic oscillations.

**Figure 6:**
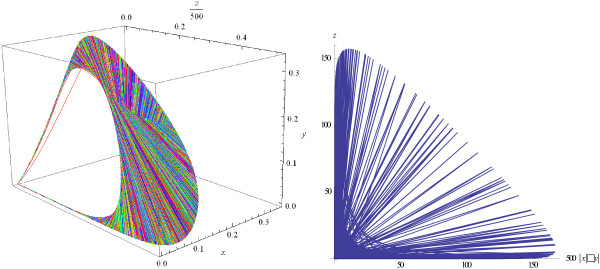
**Left panel: phase curves fill a surface, M = 500; right panel: total host population against the virus population, 1000 <** ***t*** **< 100000.**

It should be emphasized that, when the value of parameter *M* crosses the bifurcation boundary, the qualitative behavior of the system changes sharply: as *M* increases, the behavior of the system becomes more and more "chaotic", with increasing non-regularity of the shapes of the trajectories (Figure 5). At large *M*, phase curves fill a surface in the (*x*, *y*, *z*) -space (see Figure 6, left panel, in contrast to Figure 4), and the phase portrait of the system reveals sharp, quasi-chaotic oscillations (see Figure 6, right panel).

## Discussion

The bifurcation analysis of the virus-host system described here reveals complex, and in particular quasi-chaotic, oscillation regimes that so far have not been previously observed experimentally or in agent-based models of the CRISPR-mediated adaptive immunity [26,28-35]. The patchy distribution of the CRISPR-Cas systems across the microbial diversity, and even within relatively narrow groups of bacteria [10,42,43], implies complex dynamics of virus-host coevolution. Here we show that, in order to detect such non-trivial co-evolutionary regimes, the model has to be sufficiently complex, or put another way, less unrealistic than toy models that deal with a single host population and a simple dependence (or independence) of immunity on the amount of the virus, such as the two-component models examined here. The key factors for the appearance of quasi-chaotic oscillations are the non-linear dependence of the immunity on the amount of viruses and the three-dimensional phase space of the model which divides the hosts into the immune and susceptible populations, so that the system consists of three components (Table 1). In a conceptually similar manner, chaotic behavior has been observed in a model of bacteriophage-host interaction where the complexity of the system is introduced through inclusion of the time-delayed formation of consumable bacterial debris as a result of cell lysis [44,45].

**Table 1:** The analyzed models of the coevolution between viruses and CRISPR-Cas-carrying hosts

**Two-component model**		
	**Malthusian**	**Logistic**
***p *** **= const**	Center *M* > *p*/(1-*p*)	Damped oscillations *M* > *p*/(1-*p*)
***p *** **=** ***p*** **( *z *)**	No stable equilibrium *z* > 0	Damped oscillations *M* > M* (finite or infinite basin of attraction)
**Three-component model**		
	**Malthusian**	**Logistic**
***p *** **= const**	Damped oscillations *M* > *p*/(1-*p*) or *s*/(1-*s*) < *M* < *p*/(1-*p*) and *l* > *l*(*e*)	Stable equilibrium for $M>\frac{\text{ad}+\text{bs}}{b\left(1-s\right)}$
***p *** **=** ***p*** **( *z *)**	Quasi-chaotic oscillations at all *M*	Damped oscillations at *M* < *M*^*cr*^; periodic oscillations at *M* ≥ *M*^*cr*^; quasi-chaotic oscillations at *M* > > *M*^*cr*^

It should be emphasized that the three-component models require the immune host to persist in the population, hence the results obtained here are only applicable to adaptive immunity that involves stable inheritance, i.e. the Lamarckian mode of evolution [13]. The CRISPR-Cas systems that function by introducing unique, directed modifications into the host genomes present the most straightforward case of such Lamarckian immunity [13,34]. Nevertheless, other adaptive immunity systems, in particular the piRNA mechanism of transposon restriction in the animal germline, function on similar principles [46]. Moreover, the siRNA branch of eukaryotic RNA interference, which is nearly universal among eukaryotes, also encompasses a substantial heritable component, albeit in this case via the epigenetic route [37,47-49]. The quasi-chaotic regime of virus-host coevolution described here might be relevant for these immunity systems as well.

Both the Malthusian and the logistic versions of the three-component model demonstrate quasi-chaotic oscillations but they appear via distinct mechanisms (Table 1). The Malthusian version has no stable modes, with unstable equilibria. For a wide area of the parameter values, the trajectories lie in a bounded domain, and for this reason, these trajectories demonstrate (quasi)chaotic behavior for all reasonable values of the virus birth rate *M*. In contrast, the logistic model has stable modes. There exists a critical value, namely a threshold of the virus birth rate *M*^*cr*^ at fixed values of all other parameters, such that the system tends to a stable non-trivial equilibrium at *M* < *M*^*cr*^. When *M* > *M*^*cr*^, the qualitative behavior of the system changes sharply. A limit cycle and (almost) periodic oscillations appear. With the further increase of *M*, the limit cycle turns into a "surface", and the behavior of the system becomes more and more "chaotic", with increasing non-regularity of the shapes of the trajectories. When *M* > > *M*^*cr*^, the system transits to a regime of quasi-chaotic oscillations.

Thus, the models described here make concrete, quantitative predictions that can be directly tested using an experimental set-up for virus-host coevolution [27,50].

## Conclusions

Comparative genomics as well as direct experiments reveal notable evolutionary instability of the CRISPR-Cas systems [36,50-52]. It is common for closely related isolates of bacteria or archaea to differ with respect to the presence or absence of CRISPR-Cas, indicating that this immune system is easily lost and gained via HGT. Rearrangements of the CRISPR-Cas loci are also extremely widespread among microbes [43,53]. Given this evolutionary plasticity, complex and in particular quasi-chaotic regimes of virus-host coevolution revealed here appear to be plausible and potentially important for the evolutionary outcomes. In light of the current, rapidly increasing interest in CRISPR research, experimental validation of such regimes could be a realistic prospect.

## Methods

### Two-component model with *p* = *const*

The host-virus dynamics may be considered within the framework of the Volterra-type models

(M1) $$\begin{array}{l} x`=x(1-\text{ax}-\mathrm{bz}\left(1-p\right))\equiv P(x,z),\\ z`=z(-d-\text{bxp}+\text{bMx}\left(1-p\right))\equiv Q(x,z)\\ \;x\geq 0,\;z\geq 0;a\geq 0,b,d,M>0 \end{array}$$

If the parameter *p* is constant and *a =* 0 then model (M1) is Hamiltonian (conservative) with the Hamiltonian *G*(*x*, *z*) = ln |*z*| + *d* ln |*x*| - *b*(*M*(1 - *p*) - *p*)*x* - *b*(1 - *p*)*z*. If $0<p<\frac{M}{M+1}$ then the system has a saddle in the origin *O* and a center in the equilibrium

$$B\left(\bar{x}=\frac{d}{b\left(M\left(1-p\right)-p\right)},\bar{z}=\frac{1-a\bar{x}}{b\left(1-p\right)}\right).$$

If *a* > 0 (logistic case), then for constant $0<p<\frac{M}{M+1}$ system (M1) has a saddle in the origin, equilibria *A*(1/*a*, 0) and $B\left(x=\frac{d}{b\left(M\left(1-p\right)-p\right)},z=\frac{b\left(M\left(1-p\right)-p\right)-\text{ad}}{b^{2}\left(1-p\right)\left(M\left(1-p\right)-p\right)}\right);$ equilibrium *B* belongs to the positive quadrant (*x*, *z*) if *b*(*M*(1 - *p*) - *p*) - *ad* > 0. In this case, *B* is a stable node/spiral and *A* is a saddle , *A* is a stable node if *B* is not positive (see, e.g., [54]).

### Two-component model with *p* = *p*(*z*)

*Malthusian version of the model (M1) (a* = 0)*.*

This system has equilibrium in the origin (0,0), which is a saddle; *z* -coordinate of any other equilibrium $\left(\bar{x},\bar{z}\right)$ has to be a root of the equation 1 - *bz*(1 - *p*(*z*)) = 0 and $\bar{x}=\frac{d}{b\left(M-p\left(\bar{z}\right)\left(1+M\right)\right)}.$ The point $\left(\bar{x},\bar{z}\right)$ is positive only if $p\left(\bar{z}\right)<M/\left(1+M\right).$

**Proposition 1**. *If p*_*z*_(*z*) < 0, *then the Malthusian model has only one non-trivial equilibrium*$B\left(\bar{x},\bar{z}\right),$*which is an unstable node/spiral for every parameter values.*

*Proof.* The Jacobian of the system in the equilibrium $\left(\bar{x},\bar{z}\right)$ is

$$J\left(\bar{x},\bar{z}\right)=\left(\begin{array}{cc} 0 & b\bar{x}(-1+p\left(\bar{z}\right)+\bar{z}p_{z}(\bar{z}))\\ -b\bar{z}(-M+\left(1+M\right)p(\bar{z})) & -b\left(1+M\right)\bar{x}\bar{z}p_{z}(\bar{z}) \end{array}\right),$$

and its determinant and trace are:

(M2) $$\begin{array}{l} \text{Det}(J\left(\bar{x},\bar{z}\right))=b^{2}\bar{x}\bar{z}(M-(1+M)p(\bar{z}))(1-p(\bar{z})-\bar{z}p_{z}(\bar{z})),\\ \begin{array}{l} \text{Trace}(J\left(\bar{x},\bar{z}\right))=-b\left(1+M\right)\bar{x}\bar{z}p_{z}(\bar{z}) \end{array}. \end{array}$$

If the function *p*(*z*) decreases monotonically, i.e. *p*_*z*_(*z*) < 0, then *p*(*z*) and monotonically increasing function $h\left(z\right)=1-\frac{1}{\text{bz}}$ can intersect only once. Thus, equation 1 - *bz*(1 - *p*(*z*)) = 0 has only one root $\bar{z}$, and the Malthusian model has only one non-trivial equilibrium $B\left(\bar{x},\bar{z}\right).$ Next, $\text{Det}\left(J\left(\bar{x},\bar{z}\right)\right)>0,\text{Tr}\;\left(J\left(\bar{x},\bar{z}\right)\right)>0$ for positive $\bar{x},\bar{z}$ if *p*_*z*_(*z*) < 0. Thus $B\left(\bar{x},\bar{z}\right)$ is unstable node or unstable spiral.

Logistic version of model (M1) (a = 1).

Equilibria of system (M1) with *a* = 1 and  *p* = *p*(*z*) defined by (3) are the points (0,0), *A*(1,0), and $B\left(\bar{x},\bar{z}\right)$ where coordinates $\bar{x},\bar{z}$ solve the system

(M3) $$\begin{array}{l} \bar{x}=\frac{d}{b\left(M-p\left(\bar{z}\right)\left(1+M\right)\right)},1-\bar{x}-b\bar{z}\left(1-p\left(\bar{z}\right)\right)=0,\\ p\left(\bar{z}\right)<M/\left(M+1\right). \end{array}$$

Denote $h\left(z\right)\equiv \frac{d-\text{bM}+b^{2}\text{Mz}}{b\left(-1-M+\text{bMz}\right)},$ then $\bar{z}$ is a root of the equation *p*(*z*) = *h*(*z*). Solutions of this equation are the points of intersection of the curves *p*(*z*) and *h*(*z*); up to two equilibrium points $B_{1}\left({\bar{x}}_{1},{\bar{z}}_{1}\right),B_{2}\left({\bar{x}}_{2},{\bar{z}}_{2}\right)$ can appear in the model with parameter variations. Denote *J*(*x*, *z*) the Jacobian of system (M1), (3) with *a =* 1:

$$J\left(x,z\right)=\left(\begin{array}{cc} 1-2x-\mathrm{bz}(1-p\left(z\right)) & -\mathrm{bx}(1-p\left(z\right)-zp_{z}(z))\\ -\mathrm{bz}(-M+\left(1+M\right)p(z)) & -d+\mathrm{bMx}(1-p\left(z\right))-\mathrm{bxp}(z)-b(1+M)\mathrm{xz}p_{z}(z) \end{array}\right),$$

$$\begin{array}{l} J\left(0,0\right)=\left(\begin{array}{cc} 1 & 0\\ 0 & -d \end{array}\right),\\ J\left(1,0\right)=\left(\begin{array}{cc} -1 & -b(1-p\left(0\right))\\ 0 & -d+\text{bM}(1-p\left(0\right))-\text{bp}(0) \end{array}\right)=\left(\begin{array}{cc} -1 & 0\\ 0 & -d-b \end{array}\right). \end{array}$$

Thus *O*(0,0) is a saddle and *A*(1,0) is a stable node for all parameter values.

Now let us consider the system consisting of equation (M3) supplemented with the equation $\text{Det}\left(J\left(\bar{x},\bar{z}\right)\right)=0.$ In the case $\lambda_{1}=\text{Trace}\left(J\left(\bar{x},\bar{z}\right)\right)\neq 0$ this system defines coordinates of saddle-node point $B\left(\bar{x},\bar{z}\right)$ whose second eigenvalue *λ*_2_ = 0. The last system supplemented with the equation $\text{Trace}\left(J\left(\bar{x},\bar{z}\right)\right)=0$ defines an additional degeneration in the system in the point $B\left(\bar{\bar{x}},\bar{\bar{z}}\right)$ where *λ*_1_ = *λ*_2_ = 0.

***Lemma 1.*** *For a wide range of fixed parameters b*, *d*, *s there exist a point*$\left(\bar{M},\bar{k}\right)$*in the* (*M, k*) -*parameter plane such that the Bogdanov-Takens bifurcation of co-dimension 2 is realized in the model (M1), a = 1 under variations of M and k close to*$\left(\bar{M},\bar{k}\right).$*The values*$\left(\bar{M},\bar{k}\right)$ and coordinates of the equilibrium $B\left(\bar{\bar{x}},\bar{\bar{z}}\right)$*where*$\;\bar{\bar{x}}=x\left(\bar{M},\bar{k}\right),\bar{\bar{z}}=z\left(\bar{M},\bar{k}\right)$*are defined by the system consisting of equations (M3) and equations*$\text{Det}\left(J\left(\bar{x},\bar{z}\right)\right)=0$*,*$\mathrm{Trace}\left(J\left(\bar{x},\bar{z}\right)\right)=0$*(see, e.g. ,*[41]*).*

We have found this bifurcation for some reasonable fixed values of the parameters *d*, *b*, *s* with the help of computer package LOCBIF [55]. Using the Lemma and Proposition 1 we prove the following statement.

**Proposition 2.** *(1) System (M1), (3) has equilibria: the saddle O*(0,0) *and stable-node A*(1, 0) *for all positive values of the parameters b*, *d*, *M*, *k* = 1, *s*;

(2) there exists positive M* *such that*

a) *for M* < M* *the system has only the equilibria O and stable equilibrium A;*

b) *for  M* > M* *the system has two more equilibria, a saddle*$B_{1}\left({\bar{x}}_{1},{\bar{z}}_{1}\right)$*and a stable topological node/spiral*

$$\;B_{2}\left({\bar{x}}_{2},{\bar{z}}_{2}\right);$$

c) there exist M^* *^ > M* *such that for M* > M^* *^the spiral$\;B_{2}\left({\bar{x}}_{2},{\bar{z}}_{2}\right)$*is placed inside an unstable limit cycle.*

The bifurcation diagram of the system under variation of the parameter *M* is shown in Additional file 1.

### Three-component model: proof of Statement 1

Let us formulate Statement 1 in more details:

(1) *Equilibrium O*(*x* = *y* = *z* = 0) *has eigenvalues λ*_1_(*O*) = 1, *λ*_2_(*O*) = - *d*, *λ*_3_(*O*) = 1 - *l*; *it is unstable for all parameter values;*

(2) *If a* > 0 *then equilibrium A(0,1/a,0) has eigenvalues λ*_1_(*A*) = - *l*, *λ*_2_(*A*) = - 1, $\;\lambda_{3}\left(A\right)=\frac{-\mathrm{da}+b\left(M\left(1-s\right)-s\right)}{a};$*it is stable for*$M<\frac{\mathrm{da}+\mathrm{bs}}{b\left(1-s\right)}$*and unstable for*$M>\frac{\mathrm{da}+\mathrm{bs}}{b\left(1-s\right)}.$

*Proof.* Jacobian of system (1) is of the form: *J*(*x*, *y*, *z*) = (*a*_*i*,*j*_)*i*, *j* = 1, 2, 3, where

*a*_1,1_ = 1 - *l* - *ax* - *a*(*x* + *y*) - *bz*(1 - *p*(*z*)), *a*_1,2_ = - *ax* + *esz*, *a*_1,3_ = *esy* - *bx*(1 - *p*(*z*)) + *bxzp*_*z*_(*z*); *a*_2,1_ = *l* - *ay*, *a*_2,2_ = 1 - *ay* - *a*(*x* + *y*) - *b*(1 - *s*)*z* - *esz*, *a*_2,3_ = - *b*(1 - *s*)*y* - *esy*,

$$a_{3,1}=\mathrm{bMz}\left(1-p\left(z\right)\right)-\mathrm{bzp}\left(z\right),a_{3,2}=\mathrm{bM}\left(1-s\right)z-\mathrm{bsz},$$

$$a_{3,3}=-d+b\left(M\left(x+y\right)-\left(M+1\right)\left(p\left(z\right)x+\mathrm{sy}\right)\right)-\mathrm{bxz}p_{z}\left(z\right)\left(1+M\right).$$

*p*_*z*_(*z*) = 0, if *p* = *const* and *p*_*z*_(*z*) = *k*(*s* - *p*(*z*)), if *p*(*z*) = (1 - *s*)*e*^- *kz*^ + *s*.

$$\begin{array}{l} J\left(0,0,0\right)=\left(\begin{array}{ccc} 1-l & 0 & 0\\ l & 1 & 0\\ 0 & 0 & -d \end{array}\right),\\ J\left(0,1/a,0\right)=\left(\begin{array}{ccc} -l & 0 & \mathrm{bes}/a\\ -1+l & -1 & b\left(-1+s-\mathrm{es}\right)/a\\ 0 & 0 & -(\mathrm{ad}-b\left(M\left(1-s\right)-s\right)/a \end{array}\right). \end{array}$$

So, the equilibrium *O*(0, 0, 0) is unstable for both Malthusian and logistic models, the equilibrium $A\left(0,\frac{1}{a},0\right)$ of logistic model is stable for $M<\frac{\mathrm{ad}+\mathrm{bs}}{b\left(1-s\right)}$ and unstable for $M>\frac{\mathrm{ad}+\mathrm{bs}}{b\left(1-s\right)}.$*The Statement is proven.*

### Three-component Malthusian model with constant immunity *p,* ***p*** ≥ ***s*** > 0

Let us analyze the dynamics of model (1) depending on the parameters *l*, *e*. The system has trivial unstable equilibrium *O*(0,0,0). Let firstly *p* = *s*. Then system (1) by changing of variables *u* = *x* + *y*, *z* = *z* is reduced to 2-component Malthusian system (M1) with respect to *u*, *z* - variables. For $s=p<\frac{M}{M+1}$ the orbits {*u*, *z*} of this system belongs to the positive quadrant, the non-trivial equilibrium $\bar{u}=\frac{d}{b\left(M\left(1-s\right)-s\right)},\bar{z}=\frac{1}{b\left(1-s\right)}$ is a center, i.e., it is located inside closed orbits (similar to model (M1)) and trajectories demonstrate periodic oscillations. In variables *x*(*t*), *y*(*t*), *z*(*t*) model (1) also demonstrates periodic oscillations; the model has equilibrium *B*(*x*_*e*_, *y*_*e*_,  *z*_*e*_) where

(M4) $$\begin{array}{l} x_{e}=\frac{\mathrm{des}}{b\left(M\left(1-s\right)-s\right)\left(\mathrm{bl}\left(1-s\right)+\mathrm{es}\right)},\\ y_{e}=\frac{\mathrm{dl}\left(1-s\right)}{\left(M\left(1-s\right)-s\right)\left(\mathrm{bl}\left(1-s\right)+\mathrm{es}\right)},\\ z_{e}=\frac{1}{b\left(1-s\right)} \end{array}$$

If ***p*** > ***s*** then any non-trivial equilibrium (*x*_*e*_, *y*_*e*_, *z*_*e*_) of model (1) is such that the coordinate *z*_*e*_ solves the quadratic equation

(M5) $$\left(1-l\right)+b\left(-2+l+p+s-\mathrm{es}\left(1-l\right)\right)z+b^{2}\left(1-p\right)\left(1-s+\mathrm{es}\right)z^{2}=0,$$

and coordinates *x*_*e*_, *y*_*e*_ can be expressed via *z* = *z*_*e*_ as follows:

(M6) $$x_{e}=\frac{-d\left(1-b\left(1-s\right)z\right)}{b\left(p-s\right)\left(1+M-\mathrm{bz}\right)},\;y_{e}=\frac{d\left(1-b\left(1-p\right)z\right)}{b\left(p-s\right)\left(1+M-\mathrm{bz}\right)}.$$

Let $l\left(e\right)=\left(-M+p+\mathrm{Mp}\right)\left(1+\frac{\left(s(1+M\right)}{\left(M-s(1+M\right)}e\right).$ This equation defines a boundary line *Γ* = (*e*, *l*(*e*)) in the parametric plane (*e, l*).

Proposition 3.

1) *System (1) with p = const and a = 0 has at most one positive equilibrium B*(*x*_*e*_, *y*_*e*_, *z*_*e*_) *where x*_*e*_, *y*_*e*_, *z*_*e*_*are defined by formulas* (M5), (M6) for *s* < *p* and by formula (M4) for *s* = *p;*

2) *the system has a single positive equilibrium B* (*x*_*e*_, *y*_*e*_, *z*_*e*_) *if and only if one of the following conditions holds:*

a) $s<p<\frac{M}{M+1};$

b) $s<\frac{M}{M+1}<p$ and *l* > *l*(*e*);

3) *in the case a) the equilibrium B is asymptotically stable;*

4) *for s* = *p the system demonstrates periodic oscillations of variablesx(t)*, *y*(*t*) *whilez*(*t*) *tends to a stable value for a wide domain of initial values* (*x*_**0,**_ *y*_0,_ *z*_0_) *close to the equilibrium B.*

Proof.

Taking the solutions of quadratic equation (M5) in the form

$$\begin{array}{l} z=z_{1}\left(l,e\right)=\frac{2-l-\left(p+s\right)+\mathrm{es}(1-l)+\sqrt{D}}{2b\left(1-p\right)\left(1-s+\mathrm{es}\right)},\\ z=z_{2}\left(l,e\right)=\frac{2-l-\left(p+s\right)+\mathrm{es}(1-l)+\sqrt{D}}{2b\left(1-p\right)\left(1-s+\mathrm{es}\right)} \end{array}$$

where *D* = *l*^2^(1 - *s*)^2^ + (*p* - *s* + *es*)^2^ - 2*l*(*p*(1 - *s* + 2*es*) - *s*(1 + *e* - *s* + *es*) we can easily verify that both "branches" *z*_1_(*l*, *e*), *z*_2_(*l*, *e*) are real for any positive (***e***, ***l***) because the expression under the radical is non-negative. The branches *z*_1_(*l*, *e*), *z*_2_(*l*, *e*) are positive both if  *l* < 1 and only *z*_1_(*l*, *e*) is positive if  *l* > 1.

Analysis of formulas (M5), (M6) shows that only the branch *z*_1_(*l*, *e*) can define positive coordinates of the equilibrium *x*_*e*_ = *x*(*z*_1_), *y*_*e*_ = *y*_*e*_(*z*_1_). Substituting *z*_1_(*l*, *e*) into formulas (M6) we obtain that *x*_*e*_(*e*, *l*), *y*_*e*_(*e*, *l*) are positive if the point (*e*, *l*) in the parametric plane is placed above the boundary line *Γ* given by equation

$$\begin{array}{l} \left(-1+p\right)\left(1+\left(-1+e\right)s\right)(l\left(M\left(-1+s\right)+s\right)\\ \;+\left(M\left(-1+p\right)+p\right)\left(\left(-1+e\right)s+M\left(1+\left(-1+e\right)s\right)\right)=0. \end{array}$$

Statements 1 and 2 of the Proposition are proven.

Let us analyze a stability of equilibrium *B* (*x*_*e*_, *y*_*e*_, *z*_*e*_) of the system. For *p = s* characteristic polynomial of the system in the point *B,* whose coordinates are given by (M4), is of the form $E\left(\mu_{0}\right)=\mathrm{Det}(\left(J\left(B\right)-\mu_{0}I\right)=\frac{\left(d+\mu_{0}^{2}\right)\left(b\left(l+\mu_{0}\right)\left(1-s\right)+\mathrm{es}\right)}{b\left(1-s\right)}$. Thus, two eigenvalues of the point are imaginary, $\;{\mu_{0}}^{1,2}=\pm i\sqrt{d},\;$ and the third is negative, $\;{\mu_{0}}^{3}=-\frac{\mathrm{es}+\mathrm{bl}\left(1-s\right)}{b\left(1-s\right)}.$*Thus, statement 4 is proven.*

We show now that for *p* > *s* the point *B* is a sink, i.e., its eigenvalues have negative real parts (more exactly, one eigenvalue is real negative, and two others are complex with negative real part).

Introduce the parameter *α* = *p* - *s* and write the right hands of (1), *a =* 0 in the form:

$$\begin{array}{l} P\left(x,y,z\right)=x-\mathrm{lx}-b(1-\alpha -s)\mathrm{xz}+\mathrm{esyz},\\ Q\left(x,y,z\right)=\mathrm{lx}+y-b(1-s)\mathrm{yz}-\mathrm{esyz},\\ R\left(x,y,z\right)=z(-d+\mathrm{bM}((1-\alpha -s)x+(1-s)y)\\ \;-b((\alpha +s)x+\mathrm{sy})). \end{array}$$

From the condition *R*(*x*, *y*, *z*) = 0, *Q*(*x*, *y*, *z*) = 0 we can express *x* = *x*_*e*_, *y* = *y*_*e*_ via *z* = *z*_*e*_:

$$\begin{array}{l} x_{e}=\frac{-(d\left(1-b\left(1-s\right)z+\mathrm{esz}\right)}{b(\alpha \left(1+M\right)\left(1-b\left(1-s\right)z+\mathrm{esz}\right)+\left(M\left(1-s\right)-s\right)\left(-1+l+\mathrm{esz}+\mathrm{bz}\left(1-s\right)\right)}.\\ y_{e}=\frac{\mathrm{dl}}{b(\alpha \left(1+M\right)\left(1-b\left(1-s\right)z+\mathrm{esz}\right)+\left(M\left(1-s\right)+s\right)\left(-1+l+\mathrm{esz}+\mathrm{bz}\left(1-s\right)\right)} \end{array}$$

Substituting *x* = *x*_*e*_, *y* = *y*_*e*_ to *P*(*x*, *y*, *z*) we obtain:

$$P\left(x_{e},y_{e},z\right)\equiv H\left(z\right)=\frac{d\left(l\left(1-b\left(1-s\right)z\right)-\left(1-b\left(1-s\right)z-\mathrm{esz}\right)\left(1-b\left(1-s-\alpha \right)z\right)\right)}{b(\alpha \left(1+M\right)\left(1-b\left(1-s\right)z+\mathrm{esz}\right)+\left(M\left(1-s\right)+s\right)\left(-1+l+\mathrm{esz}+\mathrm{bz}\left(1-s\right)\right)}.$$

Solving the equation *H*(*z*) = 0 we get two roots *z*_*e*1_, *z*_*e*2_; supposing *z* = *z*_0_ + *αh*_*z*_ and expanding *H*(*z*) in series by *α* we get the two roots up to *o*(*α*)  in the form:

(M7) $$\begin{array}{ll} z_{e1}=z_{01}+\alpha h_{1z}, & z_{e2}=z_{02}+\alpha h_{2z},\\ z_{01}=\frac{1}{b\left(1-s\right)}, & h_{1z}=\frac{\mathrm{es}}{b{\left(1-s\right)}^{2}\left(\mathrm{bl}\left(1-s\right)+\mathrm{es}\right)};\\ z_{02}=\frac{1-l}{b\left(1-s\right)+\mathrm{es}}, & h_{2z}=\frac{\mathrm{bl}\left(1-l\right)}{\left(b\left(1-s\right)+\mathrm{es})(\mathrm{bl}\left(1-s\right)+\mathrm{es}\right)}. \end{array}$$

Notice, that the root *z*_*e*1_ is positive, whereas *z*_*e*2_ is positive only for 0 < *l* < 1. The coordinates *x* = *x*_*e*_(*α*), *y* = *y*_*e*_(*α*) of *B* are both positive only for *z* = *z*_*e*1_.

Letting *x*_*e*1_ = *x*_01_ + *αh*_1*x*_, *y*_*e*1_ = *y*_01_ + *αh*_1*y*_, we get

(M8) $$\begin{array}{l} x_{01}=\frac{\mathrm{des}}{b\left(M\left(1-s\right)-s\right)\left(\mathrm{bl}\left(1-s\right)+\mathrm{es}\right)},\\ \;h_{1x}=\frac{-\mathrm{des}\left(e^{2}s^{2}\left(1+M\right)+b^{2}\mathrm{el}\left(1-s\right)\left(M\left(1-s\right)-s\right)+\mathrm{bel}\left(1+2M\left(1-s\right)s-2s^{2}\right)\right)}{b{\left(M\left(1-s\right)-s\right)}^{2}{\left(\mathrm{bl}\left(1-s\right)+\mathrm{es}\right)}^{3}};\\ y_{01}=\frac{\mathrm{dl}\left(1-s\right)}{b\left(M\left(1-s\right)-s\right)\left(\mathrm{bl}\left(1-s\right)+\mathrm{es}\right))},\\ \;h_{1y}=\frac{\mathrm{dels}\left(\mathrm{es}-b\left(1-s\right)\left(M-\mathrm{el}\left(1+M\right)\left(1-s\right)+s+\mathrm{Ms}\right)\right)}{b{\left(M\left(1-s\right)-s\right)}^{2}{\left(\mathrm{bl}\left(1-s\right)+\mathrm{es}\right)}^{3}}. \end{array}$$

Thus, coordinates of the equilibrium *B*_*e*_ can be presented in the form (*x*_*e*_, *y*_*e*_, *z*_*e*_) = (*x*_1*e*_ + *αh*_1*x*_, *y*_1*e*_ + *αh*_1*y*_, *z*_1*e*_ + *αh*_1*z*_), see formulas (M7), (M8).

We apply the method of small parameter expansion to verify stability of the equilibrium *B*_*e*_. Characteristic polynomial of the system can be presented in the form

$$\begin{array}{ll} \;E\left(\mu \right)=\mathrm{Det}(\left(J\left(B_{e}\right)-\mathrm{\mu I}\right) & =\frac{\left(d+\mu_{0}^{2}\right)\left(b\left(l+\mu_{0}\right)\left(1-s\right)+\mathrm{es}\right)}{b\left(1-s\right)}\\ & \;-\frac{\alpha }{b{\left(1-s\right)}^{2}\left(M\left(1-s\right)+s\right)\mathrm{bl}\left(1-s\right)+\mathrm{es})}Z\left(\mu \right) \end{array}$$

where *μ* = *μ*_0_ + *αh*_*μ*_ and

$$\begin{array}{ll} \;z\left(\mu \right) & =(-(b^{2}l(d(1+h_{\mu }(1-s))+\mu_{0}\left(\mu_{0}\left(1-3h_{\mu }\left(1-s\right)\right)\right)\\ & \;\left(+2lh_{\mu }\left(1-s\right)\right)(((1-s){}^{2}M(1-s)-s)\\ & \;-es^{2}(d+\mu_{0}(\mu_{0}+2h_{\mu }\left(1-s\right)))((1-s){}^{2}(M(1-s)-s)\\ & \;+\mathrm{be}(1-s)s(\mu_{0}(\mu_{0}(-1-3h_{\mu }\left(1-s\right))\\ & \;-4lh_{\mu }(1-s))(M(-1+s)+s)\\ & \;+d(\left(-1-h_{\mu }\left(1-s\right)\right)(M(-1+s)+s)+l(M\left(-1+s\right)\\ & \;-\mu_{0}(1+M)(-1+s)+s))))). \end{array}$$

Let now $\mu_{0}^{2}=-d.$ Substituting this value to *Z*(*μ*) and solving equation *Z*(*μ*) = 0, we find

$$h_{\mu }=\frac{\left(\mathrm{bdels}\left(M+\mu_{0}\left(1+M\right)\left(-1+s\right)-s-\mathrm{Ms}\right)\right)}{\left(2\left(M\left(-1+s\right)+s\right)\left(\mathrm{bl}\left(-1+s\right)-\mathrm{es}\right)\left(b\left(d-l\mu_{0}\right)\left(-1+s\right)+\mathrm{es}\mu_{0}\right)\right)}$$

and Re( $h_{\mu })=\frac{e\mathrm{el}s}{2{\left(1-s\right)}^{3}}\left(\frac{\mathrm{es}\left(M\left(1+s\right)-s\right)+b\left(1-s\right)\left(l\left(M\left(1+s\right)-s\right)-d\left(1+M\right)\left(1-s\right)\right)}{b^{2}d\left(M\left(1-s\right)-s\right)}-\frac{{\left(1-s\right)}^{2}}{\mathrm{es}+\mathrm{bl}\left(1-s\right)}\right)<0$for reasonable parameter values. Thus, equilibrium *B*_*e*_ is asymptotically stable for *s* < *p*.

The Proposition is proven.

### Three-component logistic model with constant immunity *p*

The logistic version of model (1) with ***p*** = ***s*** is reduced to 2-component logistic system (M1) with respect to variables ***u*** = ***x*** + ***y***, ***z***. For $s=p<\frac{M}{M+1}$ it has two equilibria, *A*(0, 1/*a*) and $B\left(u=\frac{d}{b\left(M\left(1-s\right)-s\right)},z=\frac{b\left(M\left(1-s\right)-s\right)-\mathrm{ad}}{b^{2}\left(1-s\right)\left(M\left(1-s\right)-s\right)}\right).$ According to Proposition 2, these equilibria are stable in different parameter domains.

In variables ***x***, ***y***, ***z*** the equilibrium *B*(*x*_*e*_, *y*_*e*_, *z*_*e*_) of system (1) has coordinates

(M9) $$\begin{array}{l} x_{e}=\frac{\mathrm{des}\left(b\left(M\left(1-s\right)-s\right)-\mathrm{ad}\right)}{b\left(M\left(1-s\right)-s\right)\left(b^{2}l\left(1-s\right)\left(M\left(1-s\right)-s\right)+\mathrm{es}\left(b\left(M\left(1-s\right)-s\right)-\mathrm{ad}\right)\right)},\\ y_{e}=\frac{\mathrm{bdl}\left(1-s\right)}{b\left(M\left(1-s\right)-s\right)\left(b^{2}l\left(1-s\right)\left(M\left(1-s\right)-s\right)+\mathrm{es}\left(b\left(M\left(1-s\right)-s\right)-\mathrm{ad}\right)\right)},\\ z_{e}=\frac{b\left(M\left(1-s\right)-s\right)-\mathrm{ad}}{b^{2}\left(1-s\right)\left(M\left(1-s\right)-s\right)}. \end{array}$$

Let *p* be a constant, *p* > *s* > 0 . According to Statement 1, the system has trivial equilibrium *O*(*x* = 0, *y* = 0, *z* = 0), which is unstable for all parameter values, and the equilibrium $\;A\left(x=0,y=\frac{1}{a},z=0\right),$ which is stable if $M<\frac{\mathrm{ad}+\mathrm{bs}}{b\left(1-s\right)}$ and unstable if $M>\frac{\mathrm{ad}+\mathrm{bs}}{b\left(1-s\right)}.$ The system has also a non-trivial equilibrium *B* whose *x*, *y* - coordinates are expressed via *z* -coordinate:

(M10) $$\begin{array}{l} x=\frac{-d+bf^{\pm }\left(z\right)\left(M\left(1-s\right)-s\right)}{b\left(1+M\right)\left(p-s\right)},\\ y=\frac{d-bf^{\pm }\left(z\right)\left(M\left(1-p\right)-p\right)}{b\left(1+M\right)\left(p-s\right)},\text{where}\\ f^{\pm }\left(z\right)=\frac{1+M-\mathrm{bz}\pm \sqrt{{\left(1+M-\mathrm{bz}\right)}^{2}-4\mathrm{ad}\left(1+M\right)z}}{2a\left(1+M\right)} \end{array}$$

and *z* -coordinate solves the equation:

(M11) $$\begin{array}{l} \left(-1+l+af^{\pm }\right)(d-bf^{\pm }(M(1-s)-s))+(\mathrm{des}-b^{2}f^{\pm }(1-p)(M(1-s)-s)\\ \;+b(d\left(1-p\right)-ef^{\pm }(M(1-p)-p)s))z=0. \end{array}$$

Denote

$$\begin{array}{l} h^{\pm }\left(z\right)\equiv \frac{\left(d\left(l-1+af^{\pm }\right)+bf^{\pm }\left(M\left(1-af^{\pm }-l\left(1-s\right)\right)+\mathrm{ls}\right)\right)+\left(d-bf^{\pm }M\right)\left(b\left(1-s\right)+\mathrm{es}\right)z}{bf^{\pm }\left(1+M\right)\left(1-af^{\pm }-\left(b\left(1-s\right)+\mathrm{es}\right)z\right)} \end{array}$$

then $\bar{z}$ is a root of the equations *p* = *h*^±^(*z*). Solutions of the latter equation are the points of intersection of the line 0 < *p* ≤ 1 and the curves *h*^±^(*z*). Two cases with different values of the parameter *M* are presented in Additional file 2. It demonstrates that at most two positive values of $\bar{z}$ are possible and hence the system may have at most two positive equilibria, whose *x*, *y* ‒ coordinates are expressed via $\bar{z}$ by the equations (M9).

Let us define the critical value of the parameter *M*, $M^{\mathrm{tc}}=\frac{\mathrm{ad}+\mathrm{bs}}{b\left(1-s\right)}.$

**Proposition 4.** *For*$s\leq p<\frac{M}{M+1}$*and fixed positive values of  d*, *b* < 1, *l*, *e system (1) with a =* 1 *has a single stable equilibrium*$\;A\left(x=0,y=\frac{1}{a},z=0\right)$*if M* < *M*^*tc*^*and a single stable equilibrium B*(*x*_0_, *y*_0_, *z*_0_) *if M* > *M*^*tc*^; *here the coordinates* (*x*_0_, *y*_0_, *z*_0_) *are defined by formulas (M10, M11) if s < p and by (M9) if s = p. Transcritical bifurcation "changing of stability" of the points A and B happens as M* = *M*^*tc*^.

For *p* = *s* system (1) has nontrivial point *B,* whose coordinates are expressed by (M9); it is easily to verify that $u_{e}=x_{e}+y_{e}=\frac{d}{b\left(M\left(1-s\right)-s\right)},z_{e}=\frac{b\left(M\left(1-s\right)-s\right)-\mathrm{ad}}{b^{2}\left(1-s\right)\left(M\left(1-s\right)-s\right)},$ and 1 - *a*(*x*_*e*_ + *y*_*e*_) - *b*(1 - *s*)*z*_*e*_ = 0. Hence, *z*_*e*_ > 0 if > *M*^*tc*^; at this condition the equilibrium *A* looses stability according to Statement 1.

Characteristic equation at the equilibrium *B* for *p* = *s* is of the form:

$$\;\mathrm{Det}(\left(J\left(B\right)-\mathrm{\mu I}\right)=-\left(-1+l+\mu +a\left(x_{e}+y_{e}\right)+b\left(1-s\right)z_{e}+{\mathrm{sz}}_{e}\right)*$$

$$\left(\mu^{2}+b^{2}\left(1-s\right)\left(M\left(1-s\right)-s\right)\left(x_{e}+y_{e}\right)z_{e}+\mu \left(-1+2a\left(x_{e}+y_{e}\right)+{\mathrm{bz}}_{e}\left(1-s\right)\right)\right)=0.\;$$

Accounting the equality 1 - *a*(*x*_*e*_ + *y*_*e*_) - *b*(1 - *s*)*z*_*e*_ = 0, we obtain from the first term of the last equation: *μ*_1_ = 1 - *l* - *au*_*e*_ - *b*(1 - *s*)*z*_*e*_ - *esz*_*e*_ = - *l* - *sz*_*e*_ < 0, and from the second term $\mu_{2,3}=\frac{1}{2}\left(-\mathrm{au}\pm \sqrt{-4\left(b\left(M\left(1-s\right)-s\right)-\mathrm{ad}\right)u_{e}+{\left(\mathrm{au}\right)}^{2}}\right).$ For = *M*^*tc*^*μ*_2_ = 0, *μ*_3_ = - *au* < 0. Hence, the transcritical (pitchfork) bifurcation happens with points *A*, *B*[41], ch.7. When *M* > *M*^*tc*^ the eigenvalues *μ*_2,3_ have negative real parts. So, the equilibrium point *B* is stable for *p = s* and *M* > *M*^*tc*^.  Due to continuity arguments the point *B* is also stable for *p* > *s* if *p* is close to *s.* A general case *p* > *s* was verified by computation of eigenvalues of complete characteristic equation in *B*.

### Three-component Malthusian model with the immunity *p* = *p*(*z*); proof of Statement 2

The coordinates of non-trivial equilibria are given by formulas (M5) and (M6). Solving equation (M5) with respect to *p* = *p*(*z*), we obtain the equation for *z*-coordinate:

(M12) $$\begin{array}{ll} \;p\left(z\right)\equiv \left(1-s\right)e^{-\mathrm{kz}}+s & =\frac{-\left(1-l\right)+\left(b\left(2-l\left(1-s\right)-s\right)+\mathrm{es}\right)z-b\left(b\left(1-s\right)+\mathrm{es}\right)z^{2}}{\mathrm{bz}\left(1-\left(b\left(1-s\right)+\mathrm{es}\right)z\right)}\\ & \;\equiv h\left(z\right) \end{array}$$

Evidently, the *z*-coordinate of possible equilibrium does not depend on the parameter *M*. Next, *h*(*z*) → 1, *p*(*z*) → *s* < 1 *as z* → ∞, so that two general cases of mutual placing of *p*(*z*) and *h*(*z*) for *l* > 1 and *l* < 1 are possible, which are shown in Additional file 3. Equation (M12) has up to two ("small") positive roots *z* = *z*_*e*_ if *l* < 1 (see Additional file 3a) and one ("large") positive root *z*_*e*_ if *l* > 1  (see Additional file 3b). Equilibrium values *x*_*e*_(*z*), *y*_*e*_(*z*) defined by the formulas (M6) have different signs for small values of *z*_*e*_ and are both positive for large *z*_*e*_. Accordingly, the system can have only one positive equilibrium. Eigenvalues of this equilibrium can be computed from the equation *Det*(*J*(*x*_*e*_, *y*_*e*_, *z*_*e*_) - *μI*) = 0. Using the LOCBIF software [55], we show that one eigenvalue is real and negative but two other are complex with a positive real part. Thus, this equilibrium is unstable. *Statement 2 is proven.*

### Three-component logistic model with the immunity *p* = *p*(*z*)

Proof of Statement 3

Let *B*_*M*_(*x*, *y*, *z*)  be a non-trivial stable equilibrium of model (1), (3) whose coordinates (*x*(*M*), *y*(*M*), *z*(*M*)) depend on the parameter *M*  and satisfy system (2); let *P*(*μ*) ≡ *Det*(*J*(*B*) - *μI*) = 0 be the characteristic polynomial of the system around  *B*_*M*_. The supercritical Hopf bifurcation, corresponding to changing of stability of *B*_*M*_ accompanied by appearance a stable limit cycle happens in the system when for some *M* a pair of eigenvalues becomes imaginary and certain conditions of non-degeneracy are fulfilled (see, for example, [41]). The Hopf bifurcation is supercritical if the first Lyapunov value becomes negative. The existence of this bifurcation in logistic version of model (1), (3) was verified using LOCBIF [55]. The program numerically finds coordinates of equilibrium with imaginary eigenvalues under variation of parameter *M* and one more parameter of the model (e.g., *e*, *l*, or *s*) for fixed values of other parameters, checks the sign of the first Lyapunov value and verifies the conditions of non-degeneracy formulated in the Hopf theorem. Applying this software we have found the parameter curves of Hopf bifurcation *e*_*H*_(*M*), *l*_*H*_(*M*); *s*_*H*_(*M*) for fixed values of other parameters of the model (see Additional file 4); it proves the assertions of the Statement.

## Reviewers’ reports

### Reviewer 1: Sandor Pongor

Comment: The CRISPR-Cas system is of high theoretical and practical interest. On the practical side it is important for designing genome engineering tools, on the theoretical side, it provides a noteworthy example of Lamarckian evolution. By inserting virus-derived spacers into CRISPR repeat cassettes, microbes preserve in their genomes the signature of viruses that attack them, which in turn they pass on to their offspring. The evolution of this system is practically intriguing and has been the subject of several agent based modeling studies. As the authors point out, agent systems can be used to model various levels of complexity, however they do not permit a full and rigorous mathematical analysis of all possible behaviors. Berezovskaya and associates present here a differential equation based model of the evolution of CRISPR-Cas based immunity. They use Lotka-Volterra type models that allow the analysis of Malthusian and logistic regimes and show that the models display complex quasi-chaotic oscillations. Such complex behaviors have not been found either by experiment or by the previous agent based mutations. The results are well underpinned and clearly discussed. The results are especially interesting example of how unexpected complexity emerges in a seemingly simple system. The text is very well written however the authors may want to check it for minor typos, e.g.:

Page 3 challenge (a virus), "in contrast as opposed" to the random, undirected mutations in the Darwinian evolutionary framework - one of them is superfluous.

Page 4 after eqn 1 host reproduction is Malthusian if a = 0 or logistic if a > 0, and in both cases. – sounds like an unfinished sentence.

Authors’ response: *We appreciate these comments. The proposed corrections have been made*.

### Reviewer 2: Sergei Maslov

Comment: The manuscript addresses a very interesting topic of the emergence of chaotic oscillations in population dynamics of phages and their bacterial hosts. To my knowledge, this topic is relatively unexplored in the literature except for "Bifurcation analysis of bacteria and bacteriophage coexistence in the presence of bacterial debris" by Ira Aviram, Avinoam Rabinovitch [44]. Authors should cite this paper and compare and contrast their results to findings of Aviram et al.

Authors’ response: *As far as we can see, the work of Aviram and Rabinovitch is relevant only in a very generic sense. In the revision, we cite this paper along with a more recent publication of the same authors, and comment on the emergence of complex behavior in these models.*

Comment: I found the paper very hard to read and understand. In its present form it is unnecessarily heavy on mathematical details and terminology and light on biological insights. I would strongly recommend a *near complete rewriting* of the text of the manuscript that would delegate unnecessary mathematical details and theorems to supplementary materials and explaining the biological consequences of main findings. For example, on page 5 authors refer to their model as "conservative". It is not at all obvious to the majority of even sophisticated computational biology readers that in a conservative system small deviations from the steady state solution do not decay back to the steady state but persist indefinitely. Whenever possible authors should avoid using mathematical jargon and explain in plain English what their parameters/assumptions mean biologically.

Authors’ response: *As per this comment and analogous comments of Dr. Kimmel (see below), the entire description of two-dimensional (or two-component, using the modified terminology), which included most of the mathematical detail, was moved to the Methods. There, we considered it appropriate to formally mathematically define "conservative systems" and other concepts that might be unfamiliar to non-specialist readers. In the main text, biological interpretations of the parameters and assumptions were provided on several occasions. We believe that these changes made the paper considerably more straightforward, and we appreciate these suggestions of the reviewers. In general, however, one has to face the fact that this is a mathematical biology paper. Further, we beg to disagree that this paper is "light on biological insights". We think that the revealed complex behavior of the co-evolving virus-host systems is a potentially important biological instant, and the reviewers do not seem to disagree. What is somewhat lacking, are direct connections to experimental results. Such relevant results could come, first, from quantitative measurements of virus diversity and CRISPR-Cas prevalence in various habitats, and second, from laboratory co-evolution experiments. We expect that such results are indeed forthcoming and hope that the present facilitates their interpretation but at this time, there is little data for direct comparison*.

Comment: Another example of the same point: on page 5 authors introduce two steady state solutions A and B but do not explain what they mean biologically: phages co-exist with bacteria in A but die off in B. And this is just one example of the lack of biological interpretation of mathematical results happening throughout the manuscript.

Authors’ response: *This specific statement has been moved to the Methods as per the suggestion of Dr. Maslov and Dr. Kimmel. The interpretation of these steady solutions given by Dr. Maslov is quite correct and thus, we presume, is obvious from the presentation. Especially in the Methods section, further clarifications seem unnecessary. On several other occasions (see below), however, we did include additional biological interpretations.*

Comment: On the same page authors cite earlier studies with empirical results confirming that the fraction of immune bacteria p directly depends on the phage population z and not on the bacterial population x. A more detailed discussion of what the empirical data actually say would be beneficial here.

Author’s response: *This discussion has been expanded and made more specific*.

Comment: In particular, I don’t expect p(T) to instantaneously trace rapidly growing phage population z(T), which seems to be a prerequisite for chaotic behavior reported in this manuscript. What would happen when a delay or time averaging is added to the model? That is to say, what would happen if p(T) is a function of z(T-t_delay) or (in a separate ariant of the model) p(T) is determined by the time-averaged value of z(T) over T:T-t_average time interval? I would be particularly interested to see if chaotic dynamics would disappear for large enough values of t_delay or t_average. What is a realistic value of t_average compared to a single generation of lytic phage growth?

Authors’ response: *These are interesting and important questions that, however, are beyond the scope of the present paper*.

Comment: I also request a small yet important change in notation: authors repeatedly refer to three-dimensional version of their model which I understood as a model with 3 spatial coordinates. Indeed, special inhomogeneity is known to play an important role in phage-bacterial interactions (see e.g. [56,57]. However, what authors meant is simply a three-species or three-component model with phages, immune hosts, and susceptible hosts. To avoid confusion the term "three-dimensional model" while mathematically correct needs to be changed to "three component model" throughout the manuscript.

Authors’ response: *We adopted this change in the revision*.

Comment: Are figures of plots in Figure 1, 2, 3, 4, 5 necessary? I personally did not learn anything from them. Figure 6, Additional files 1, 2, 3 and 4 nicely illustrate chaotic dynamic of the system. Perhaps they can be collected as multiple panels of just one figure?

Authors’ response: *We agree, figures*1*,*2*,*3*,*4*,*5*have been moved to the additional files*.

### Additional comments made in the second round of review

Comment: On the other hand, steady state and simple time-dependent solutions to Lotka-Volterra equations for bacterial-phage systems have been studied for quite some time. Some classic references such as [22,23] have been overlooked and need to be cited in the manuscript.

Authors’ response: *We agree, these are relevant references that are cited in the revised version of the present article*.

Comment: Population cycles and fixed points in modified Lotka-Volterra equations have been also considered in [58,59]. Would authors predictions of chaotic (or quasi-chaotic) behavior persist in these systems?

Authors’ response: *We do not see how to directly link these models with our approach (at least not without additional, extensive analysis) and therefore currently cannot make such predictions.*

Comment: Spatial (see e.g. [56]) and temporal [57] inhomogeneity of the environment is known to play an important role in phage-bacterial interactions. How much would it affect chaotic dynamics. These are not idle questions since they go to the heart of the question of how generic is the chaotic behavior reported in the manuscript. If authors believe they are beyond the scope of the current paper, perhaps, these questions should be mentioned in the discussion section as model generalizations/modifications that need to be performed in future studies.

Authors’ response: *we fully agree that these are among desirable generalizations of the present approach. It is another matter whether, with the addition of these non-homogeneities, the model remains tractable. On very general grounds, given that here we have shown that the pseudo-chaotic oscillations only emerge in a system with certain minimal complexity (distinguishing susceptible and immune hosts is essential), we would expect that such oscillations only become more prominent in even more complex models. However, this is obviously only a conjecture at this point, we cannot be confident before the actual analysis is done*.

Comment: Throughout the manuscript authors consider only virulent (lytic) phages. Would there be any interesting modification of predicted dynamical patterns for temperate phages?

Authors’ response: *As such, temperate phages, by definition, do not kill the host, and therefore, even if lysogenization is prevented by CRISPR-Cas, as indeed has been reported*[60]*, this seems to be irrelevant for the modeling approach described here. The situation certainly changes when it comes to prophage induction, against which CRISPR-Cas protects as well*[60]*. This case does not appear to be distinguishable from lytic infection within the approximations of the model*.

Comment: I appreciated authors following my request and renaming two/three dimensional model into two/three component model. However, on page 11 and several other places in the manuscript old notation is still being used. I recommend authors do global search for "2D", "3D", and " dimensional" in the manuscript.

Authors’ response: *This has been taken care of.*

#### Reviewer 3: Marek Kimmel

This paper addresses the issue of co-evolution of a virus an and the immune system of a host, taking into account the dynamics of the virus and two types of immune systems: susceptible and resistant. The model is inspired by a type of immune response (CRISP-Cas) in archaea and some bacteria. The dynamics is summarized by a system of 3 nonlinear ordinary differential equations (ODEs). The system seems to exhibit various dynamical regimes including some that are chaotic. This, according to the authors, provides some analogy to the known examples of the CRISP-Cas system behavior.

The paper should be reorganized before it is publishable.

Major issues

Comment: 1. The paper is written in a way which makes understanding it very difficult. A large portion of the paper is devoted to models which are inadequate in that they do not include sensitive and resistant immune systems, are therefore limited to two ODEs and cannot exhibit complicated dynamics. To make the paper readable, it should proceed directly to the point. The auxiliary models can be moved to an appendix.

Authors’ response: *This reorganization of the manuscript has been implemented as suggested*.

Comment: 2. Dynamics of the really interesting 3-ODE models is explored mostly numerically, if I understand correctly. In my opinion, more illustrative material might be provided, using the space available after removal of the 2-ODE models.

Authors’ response: *We carefully considered this suggestion but found that the comparison of Figures*2*,*3*,*4*,*5*and*6*was highly illustrative of the results for the 3-ODE models. The transitions between the outcomes depending on the parameters was made fully explicit in the revised description*.

Comment: 3. I am missing a detailed discussion of how the model is similar to experimental observations. What type of data are available? Time series are probably most desirable. Is there any information in the data regarding sensitivity to parameter change and initial conditions, which are characteristic of chaos?

Authors’ response: *As pointed out in our response to Dr. Maslov’s comments above, the experimental data are simply not ready for detailed comparison. We would be more than happy to analyze time series or cite an appropriate analysis but such experiments belong in the future*.

Comment: 4. Finally, why would this quite generic mathematical description be specific to the CRISP-Cas systems?

Authors’ response: *We never claimed that this description was specific to CRISPR-Cas. It is inspired by CRISPR-Cas but is applicable to any system of adaptive immunity with sufficiently long term memory, as pointed out both in the abstract and in the Conclusions.*

Detailed remarks

Comment: 1. Page 9. It seems x and y are each composed of a number of different "immuno-types" of host individuals. The structure will be continuous and not two-point as it is now. What will be the consequences for the dynamics? Will it not be more regular because of smoothing effect of continuity?

Authors’ response: *To the best of our understanding, x is just one type. However, y indeed can be represented as numerous "immuno-types" if immunity to different viruses is considered separately. This situation has been explored within the framework of agent-based models*[28,32]*. Under the analytic approach used here, continuous distribution of "immune-types" would inevitably make the model intractable.*

Comment: 2. Page 15. Computational analysis usually cannot "show" that an equilibrium is stable. It may be at best consistent with stability.

Authors’ response: *The revised version of the manuscript includes a comprehensive test for stability using LOCBIF. Thus, "show" (which does not mean "proven") seems appropriate.*

3. "Proposition 4" does not seem to be mathematically demonstrated. So, it is a Conjecture.

Authors response: *A sketch of the proof is given in the revision.*

## Competing interests

The authors declare that they have no competing interests.

## Authors’ contributions

FB and GPK performed the mathematical modeling; YIW, EVK and GPK analyzed the results; GPK and EVK wrote the manuscript that was read and approved by all authors.

## Supplementary Material

Additional file 1 **Bifurcation diagram for the system (2) with *a =*** **1, *b*** **= 0.1,** ***d*** **= 1,** ***k*** **= 1,** ***s*** **= 0.15; *M *** **=12 in Domain (0), *M *** **=25 in Domain (1), *M *** **=100 in Domain (2).** Click here for file

Additional file 2 **Plots of the functions *h***^**±**^**(*z*) for *l =*** **0.5 (a) and *l =*** **1.51 (b).** In both cases, *M* = 100, *d* = 1, *b* = 0.05, *s* = 0.1, *e* = 0.1. Click here for file

Additional file 3 **Plots of the functions *p*** **(*z*),** ***h*** **(*z*) for *l =*** **0.5 (a) and *l =*** **1.5 (b).** In both cases, *M* = 100, *d* = 1, *b* = 0.05 , *k* = 0.5, *s* = 0.1, *e* = 0.1. Click here for file

Additional file 4 **Parameter curves of the Hopf bifurcation; a: *e***_***H***_**(*M*)  for *l*** **= 0.1,** ***s*** **= 0.2,** ***b*** **= 0.05,** ***k*** **= 0.2,** ***l***_***H***_**(*M*) for *e*** **= 0.5,** ***s*** **= 0.2,** ***b*** **= 0.05,** ***k*** **= 0.2 b: *s***_***H***_**(*M*) for *l*** **= 0.1,** ***e*** **= 0.5,** ***b*** **= 0.05,** ***k*** **= 0.2.** Click here for file
