# Supplementary figures and images for: Pseudo-chaotic oscillations in CRISPR-virus coevolution predicted by bifurcation analysis
Source: Biol Direct. 2014 Jul 2;9:13. doi: 10.1186/1745-6150-9-13 (PMC4096434; doi:10.1186/1745-6150-9-13)

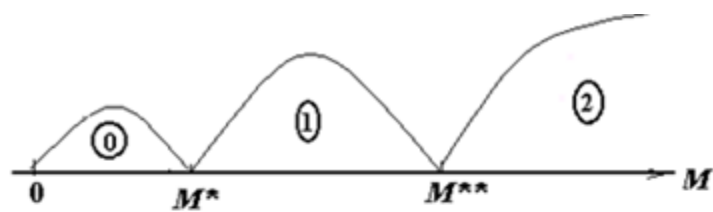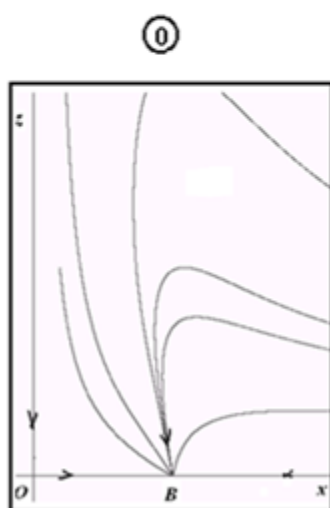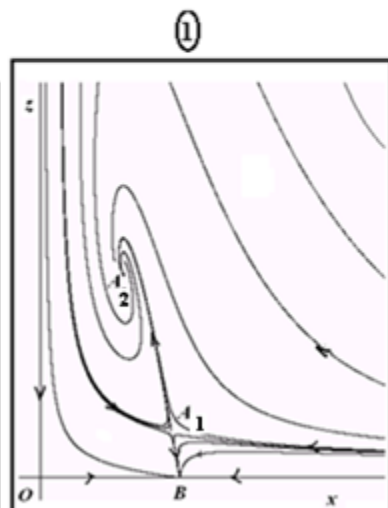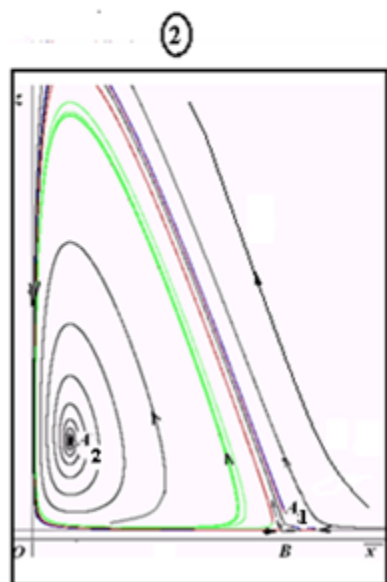

Supplement: Additional file 1 — Bifurcation diagram for the system (2) with a = 1, b = 0.1, d = 1, k = 1, s = 0.15; M =12 in Domain (0), M =25 in Domain (1), M =100 in Domain (2). [file 1745-6150-9-13-S1.pdf]

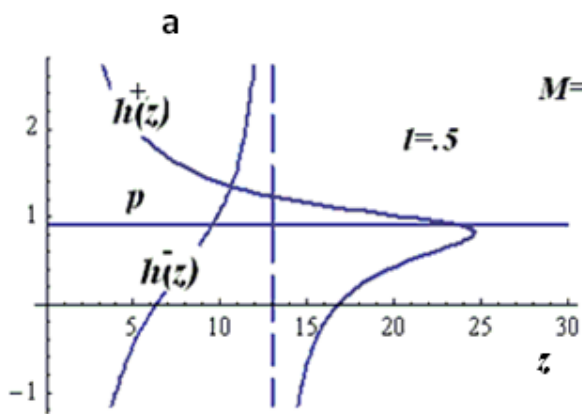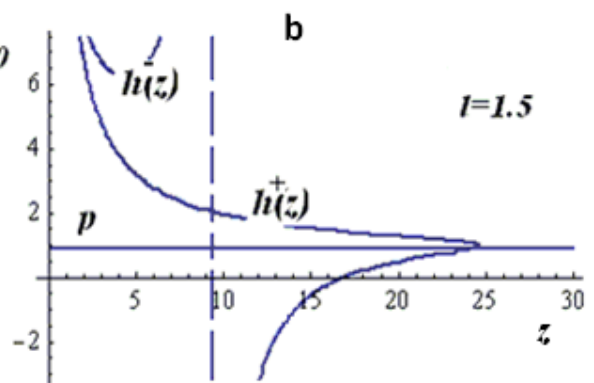

Supplement: Additional file 2 — Plots of the functions h ± ( z ) for l = 0.5 (a) and l = 1.51 (b). In both cases, M = 100, d = 1, b = 0.05, s = 0.1, e = 0.1. [file 1745-6150-9-13-S2.pdf]

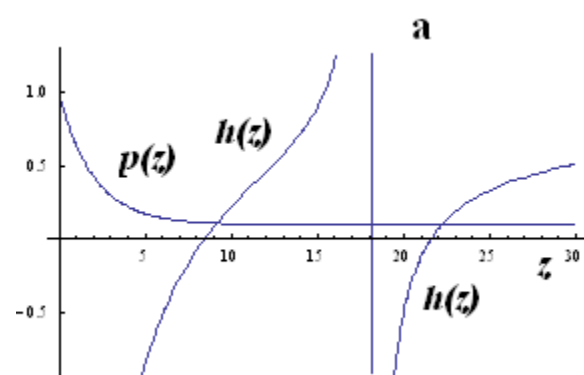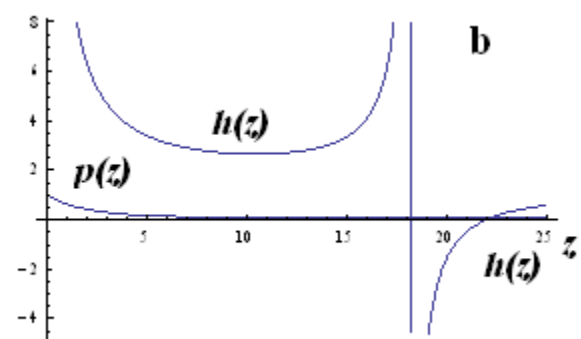

Supplement: Additional file 3 — Plots of the functions p ( z ), h ( z ) for l = 0.5 (a) and l = 1.5 (b). In both cases, M = 100, d = 1, b = 0.05 , k = 0.5, s = 0.1, e = 0.1. [file 1745-6150-9-13-S3.pdf]

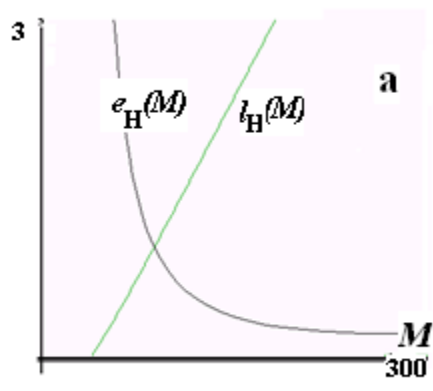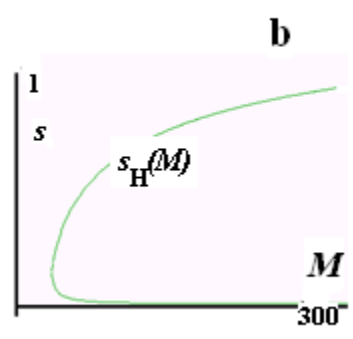

Supplement: Additional file 4 — Parameter curves of the Hopf bifurcation; a: e H ( M ) for l = 0.1, s = 0.2, b = 0.05, k = 0.2, l H ( M ) for e = 0.5, s = 0.2, b = 0.05, k = 0.2 b: s H ( M ) for l = 0.1, e = 0.5, b = 0.05, k = 0.2. [file 1745-6150-9-13-S4.pdf]
